# Supplementary material for: Gene-by-environment interactions in urban populations modulate risk phenotypes
Source: Nat Commun. 2018 Mar 6;9:827. doi: 10.1038/s41467-018-03202-2 (PMC5840419; doi:10.1038/s41467-018-03202-2)
Supplement: Supplementary file 1 — Supplementary Information [file 41467_2018_3202_MOESM1_ESM.pdf]

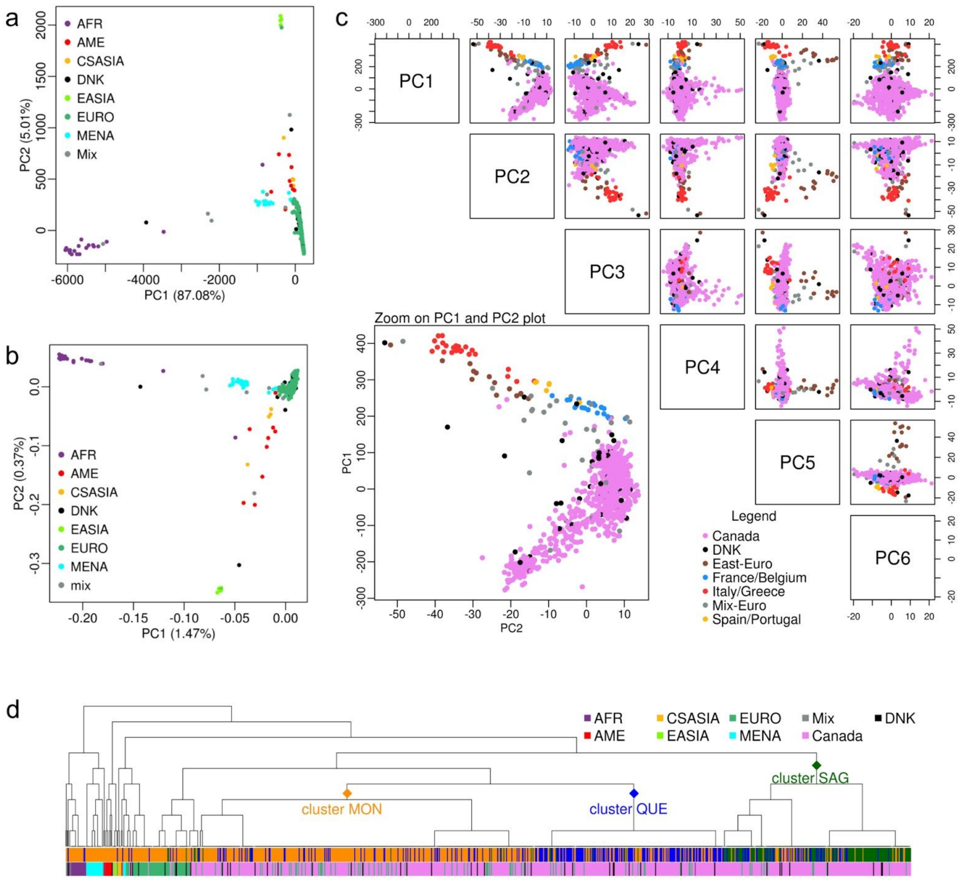

Supplementary Figure 1: Genetic structure among participants selected from the CARTaGENE cohort.

Principal component analyses on all participants, using (a) the chunk count matrix generated with ChromoPainter and (b) the LD pruned genotypes. All participants are colored according to the declared origin of the four grandparents. AFR Africa, AME America, CSASIA Central South Asia, DNK Do not know, EASIA East Asia, EURO Europe, MENA Middle East and North Africa, Mix Admixed individuals form different populations. (c) A principal component analysis on participants of European descent (French-Canadians and Europeans) on the chunkcount matrix of ChromoPainter reveals the genetic structuring present among the participants. Plots of the first six principal components, participants are colored according to the declared country of birth of the four grandparents.East-Euro includes Bulgaria, Germany, Hungary, Poland, Romania, Russia, and Ukraine. Mix-Euro indicates individuals with multiple declared countries of origin of the grandparents. (d) Tree from finSTRUCTURE highlights clusters of participants within Quebec, a structuring that is also reveals by the PCA shown in Fig. 1b and c. The first line below the tree indicates the region of residence of each participant (orange=MTL, blue=QUE, green=SAG). The second line indicates the declared ancestry of each participant.

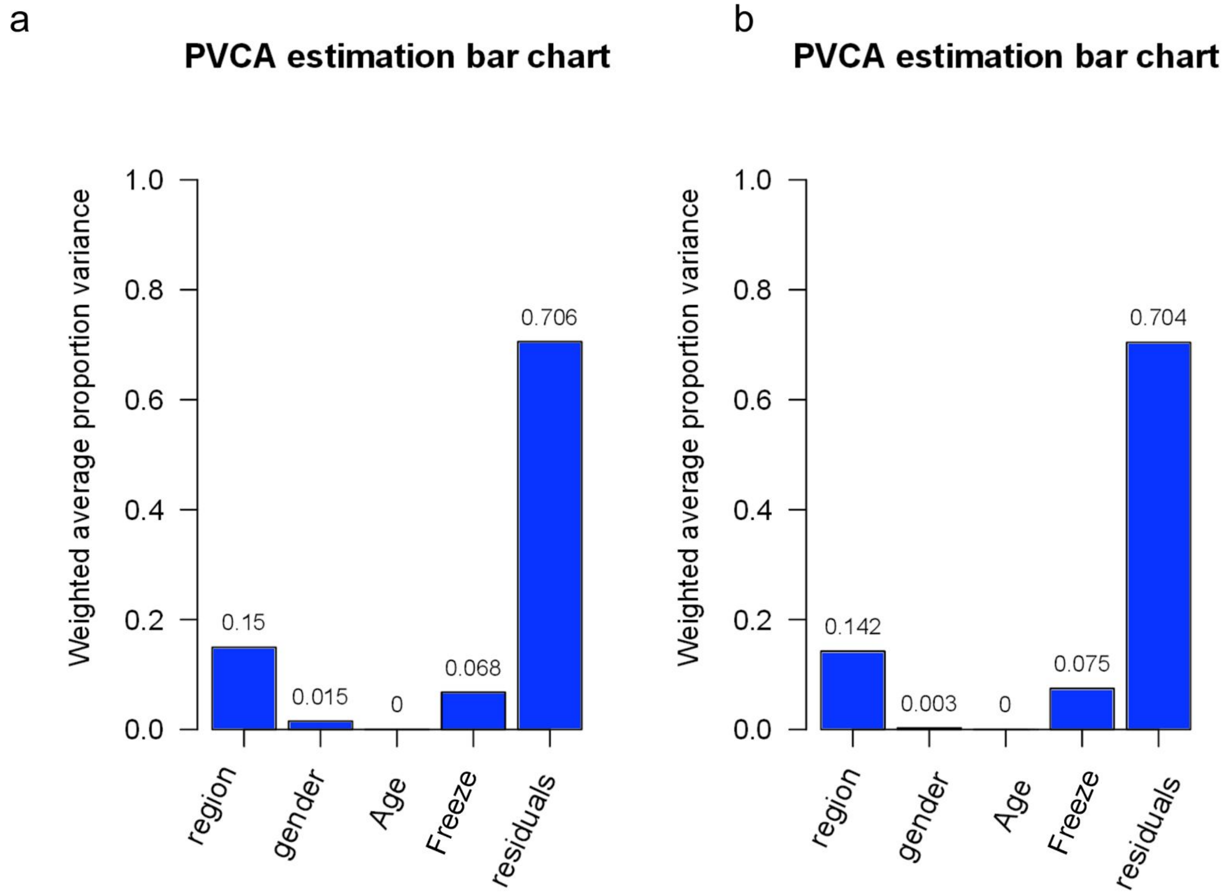

Supplementary Figure 2: PVCA for low level endophenotypes and region.
Principal Variance Component Analysis for (a) French-Canadians and (b) Europeans. In both cases, region explains around 14-15% of the variation in gene expression profiles. “Freeze” (1 or 2) are the two different batches of sequencing, and also explains an amount of the variation, therefore, freeze will be added as a covariate in downstream analyses. Note that genes with strong sex-biased expression were filtered out at QC.

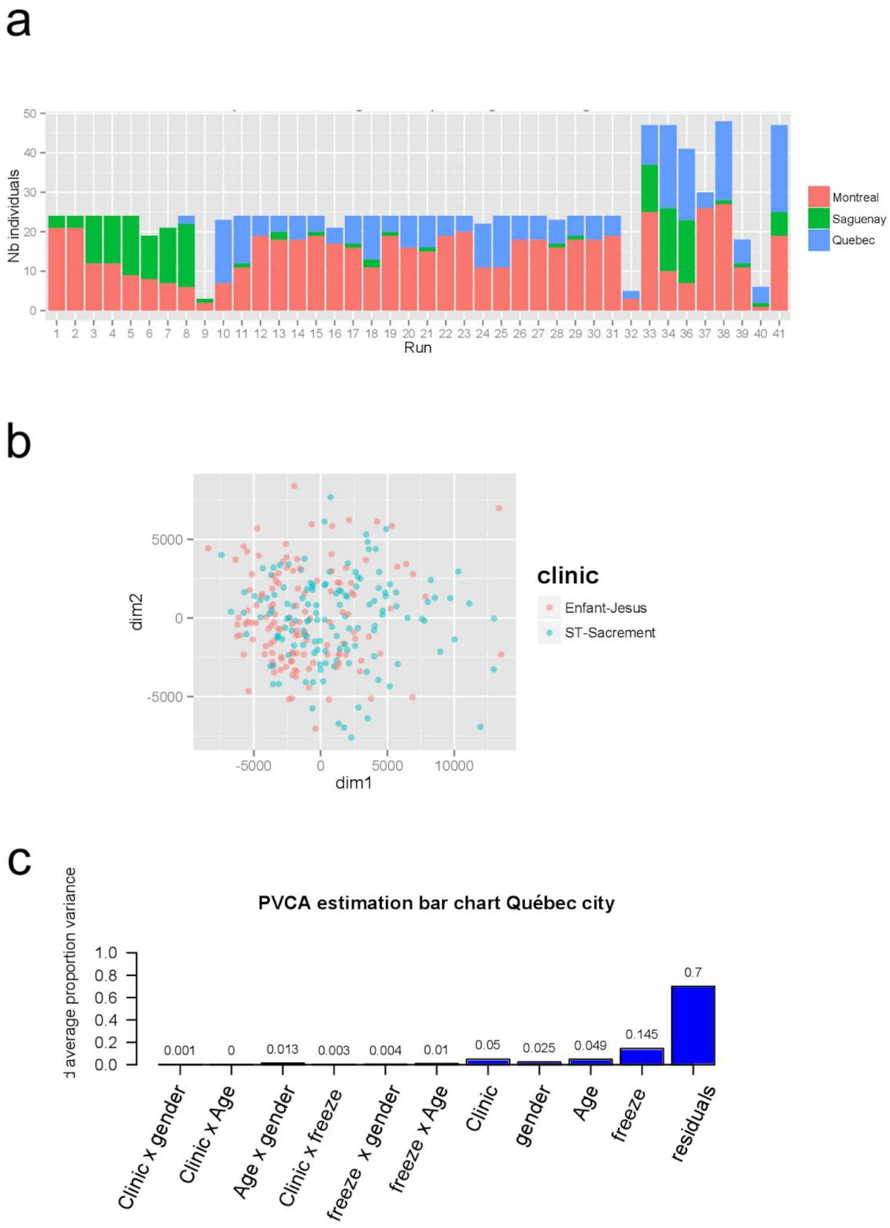

Supplementary Figure 3: RNA-Seq experimental design and batch effect assessment.
(a) The distribution of individuals from the three regions among sequencing runs shows that participants from the three regions are largely uniformly distributed among sequencing runs. (b) PCA on participants living in QUE, but for which the blood collection was performed at two different clinics within QUE region (Enfant-Jesus and St-Sacrement). There is an absence of clustering by clinic, suggesting that the blood collection center does not affect gene expression profiles within a region. (c) Similarly, a PVCA on QUE individuals only shows that clinic does not explain a large amount of the variation in gene expression.

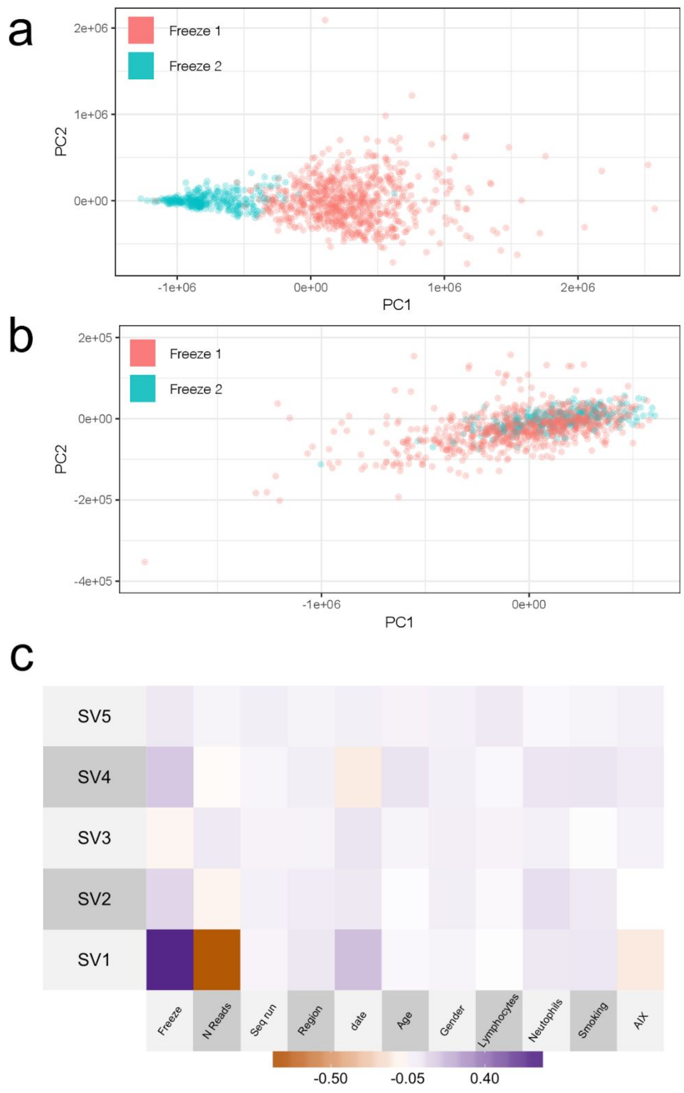

Supplementary Figure 4: The effect of SVA correction on gene expression profiles.

(a) In a principal component analysis (PCA) before correction, a very strong effect of the sequencing freeze can be observed across the dataset. (b) PCA after SVA correction (SVA 1 to 5 are the 5 surrogate variables). The effect of the Freeze is greatly reduced, although still significant (Supplementary Table 1). Other unwanted biological variation was removed by SVA (Supplementary Table 1). (c) Heatmap showing the correlation of SVA factors with several endophenotypes measured in our participants. SVA was performed to retain variation associated with region, and indeed as depicted in the heatmap, no surrogate variable show high correlation with region.

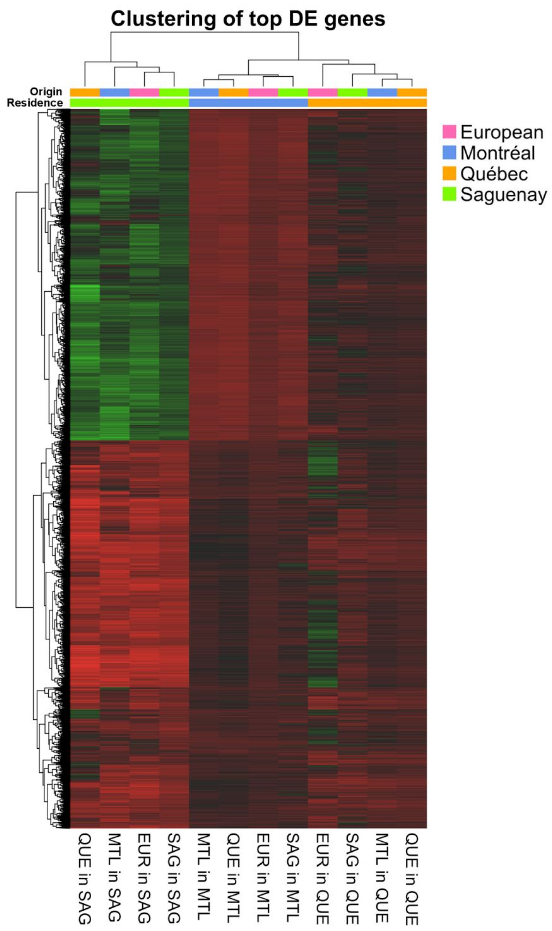

Supplementary Figure 5: Expression profiles of participants cluster by environment of residence, not ancestry.

Heatmap showing the k-means clustering of top differentially expressed genes between MTL and SAG (y-axis). The annotation at the top shows for each participant its origin (FC-regional or European), and the second one, its region of residence (environment). Participants cluster by region of residence, and not origin. We also see that participant living in MTL and QUE are more similar to each other in terms of their gene expression profiles, and that SAG participants are more divergent.

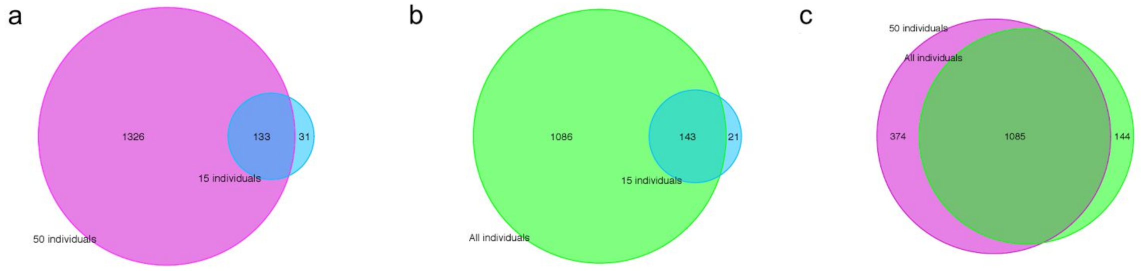

Supplementary Figure 6: Power analysis of differential expression analyses.
We show that when comparing two groups (MTL and SAG) between which we expect to find many differentially expressed genes (DEGs), a smaller sample size of either n= 15 MTL vs 15 SAG or n= 50 MTL vs 50 SAG, still allows for the detection of some DEGs. We find large overlaps of DEGs identities between these power analyses comparisons, (a) between n= 15 and n=50 analyses (b) n=15 and all individuals (MTL=234, SAG=115), and (c) between n=50 and all individuals. The largest gain in detection power of DEGs is achieved when increasing the sample size from 15 to 50. 78% of the DEGs detected with all individuals are also detected as DEGs when using n=50 individuals.

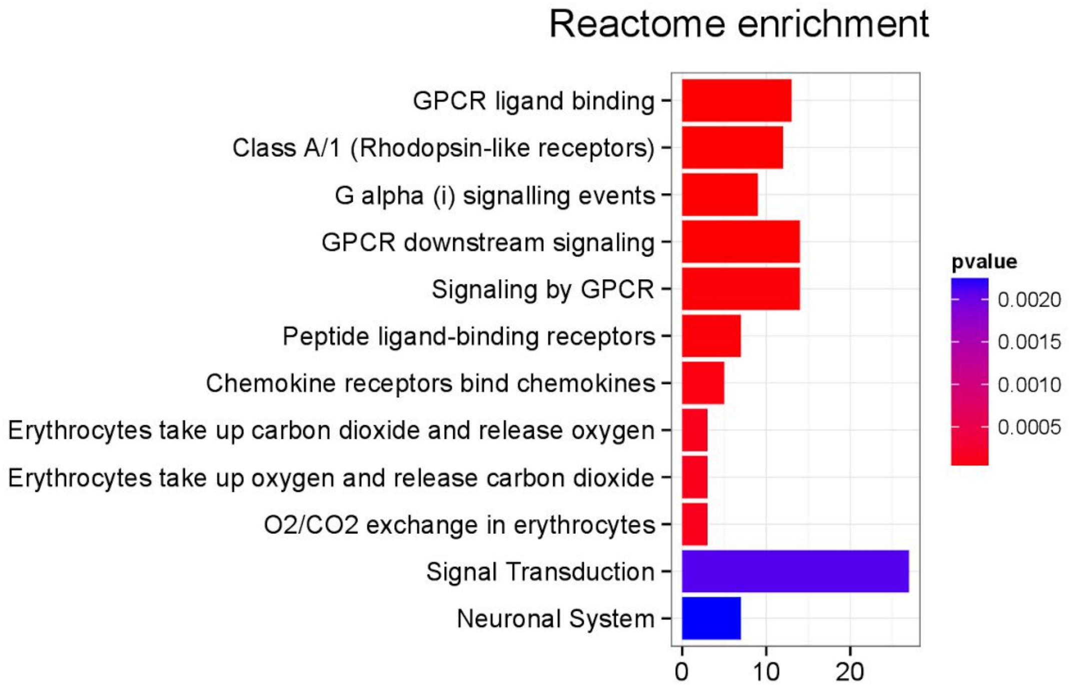

Supplementary Figure 7: Gene ontology and Reactome enrichment from top hits of differentially expressed genes between Mtl-locals and Sag-locals.

Reactome enrichment results. GO term enrichment are found in Supplementary Table 3. Gas and oxygen transport are found by both GO and reactome enrichments.

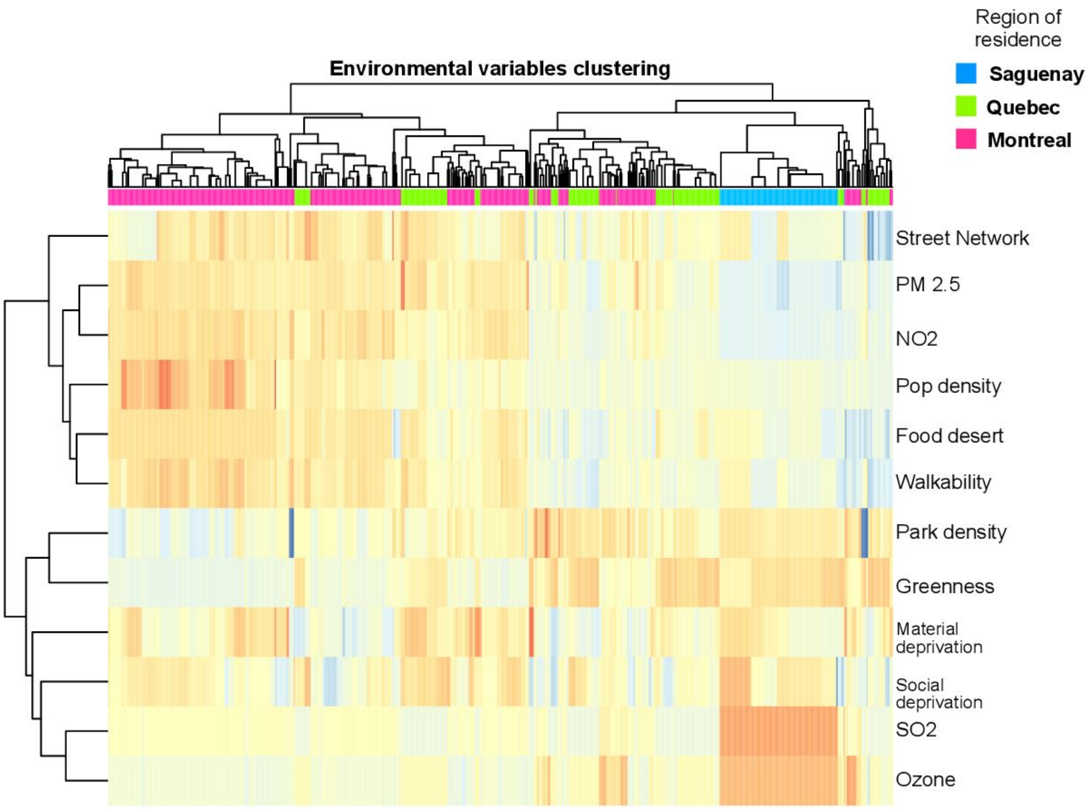

Supplementary Figure 8: Environmental variation clustering across Quebec regions shows a unique environmental profile in Saguenay.

Heatmap depicting the 12 environmental variables collated (rows) for each individual (columns). Clustering was performed on rows and columns, and the columns annotation indicates the region of residence of each individual. Saguenay individuals cluster all together, indicating a specific environmental exposure profile in Saguenay. More specifically, ozone, SO2, and NO2 levels are important in defining the specific environmental exposure profile in Saguenay. These high ambient levels in Saguenay of ozone, SO2 and NO2 has been reported recurrently by national surveys [1] and non-profit organizations [2].

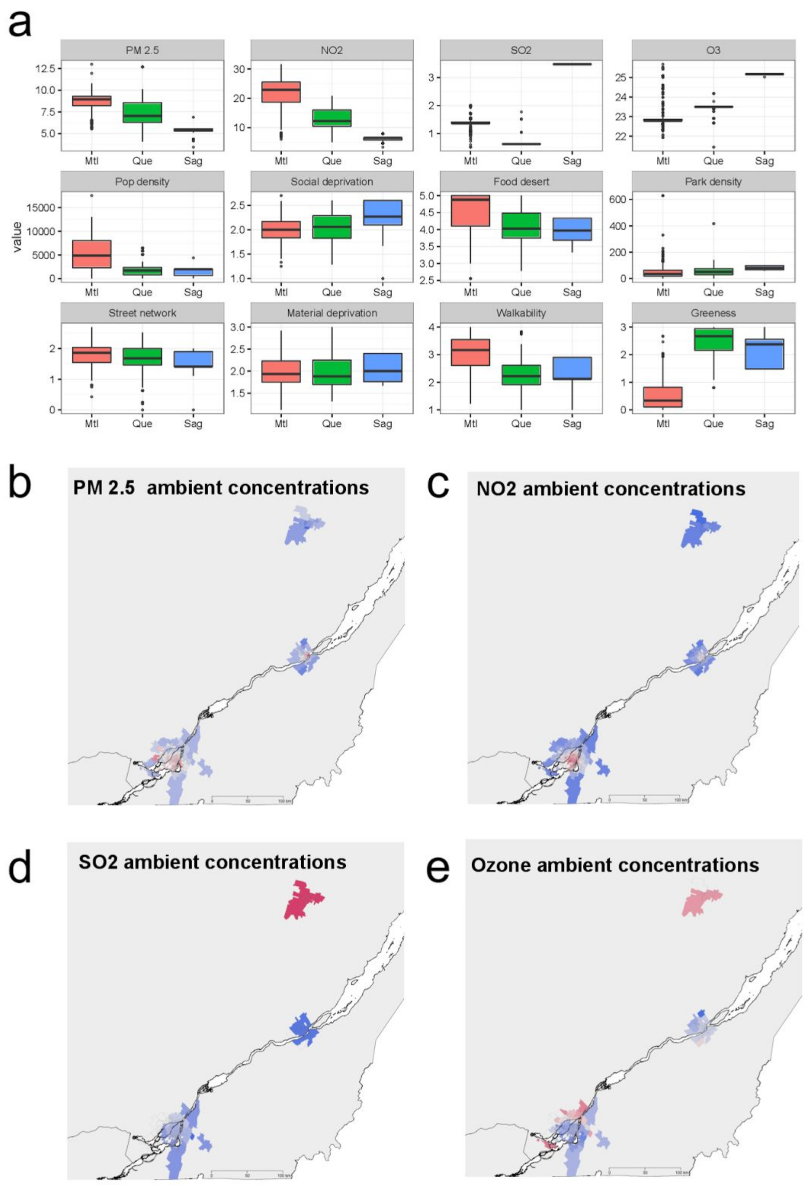

Supplementary Figure 9: Environmental variation across Quebec regions.

(a) Boxplots representing each of the environmental data for each of the three regions under study. Maps of ambient concentration across the three regions, per postal code district, of (c) SO2, (D) NO2, and (E) PM 2.5, illustrate large regional variation air composition. Color scheme represent the level of ambient concentration (parts per billion) for each pollutant (blue = lower levels, red = higher levels, see Supplementary Table 4 and material and methods for details on the data).

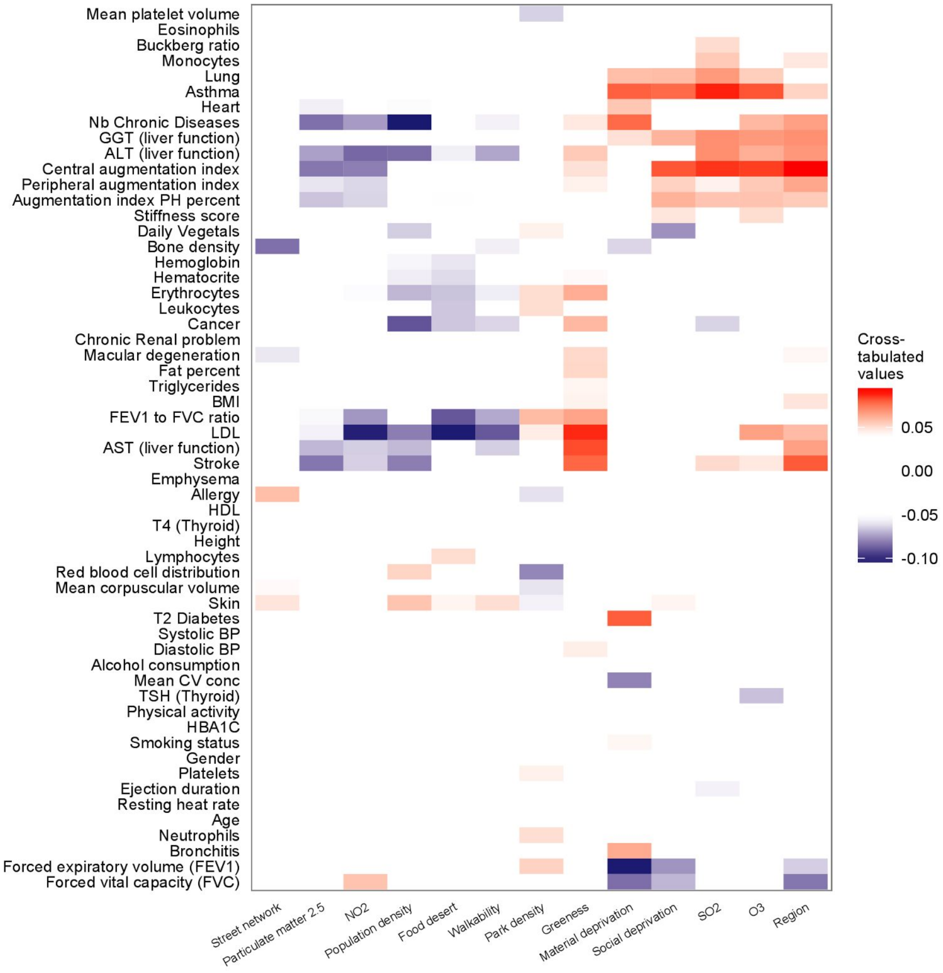

Supplementary Figure 10: Coinertia analysis (CoIA) between 57 clinically relevant endophenotypes (rows) and environmental variables (columns).

Heatmap representing the cross-tabulated values of 10 000 CoIAs based on resampling 493 individuals without replacement at each step. The median value for each endophenotype environment from the 10 000 CoIA is reported. The endophenotypes and environmental variables were clustered based on the cross-tabulated values. The environmental variable clustering largely reflect the environmental gradient across regions (Supplementary Figs 8 and 9). The strongest associations are observed between augmentation indexes (central, peripheral, and PH percent) and asthma with NO2, SO2, ozone, social deprivation, and region of residence. Liver function tests (ALT, AST, GGT) also show strong associations with several of these same environmental variables. Also, we find a negative association between FEV1 and FVC with region, with lower values of these spirometry measures in SAG. All biochemical endophenotypes were measured in a single central laboratory.

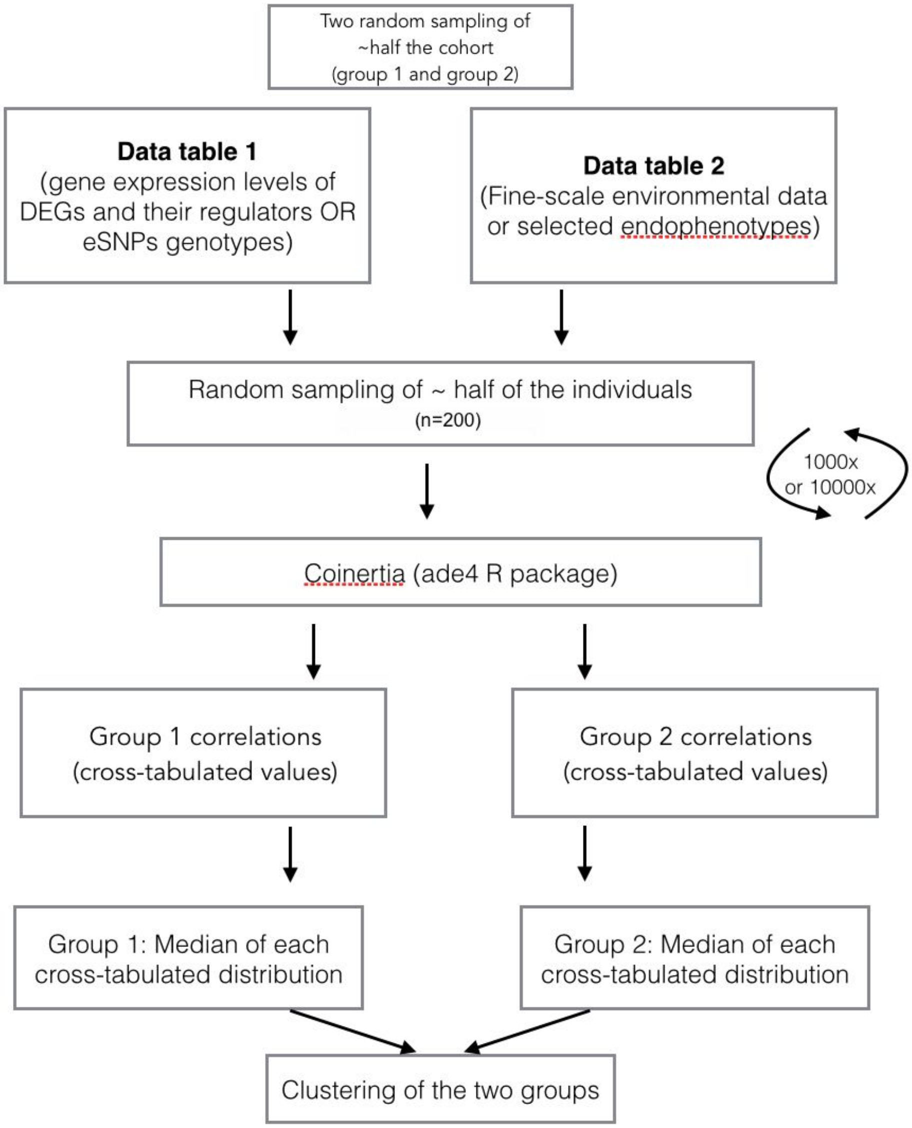

Supplementary Figure 11: Coinertia anlayses resampling scheme.

We used this resampling scheme for (1) performing the CoIA between the differentially expressed genes plus their regulators and the fine-scale environmental variables (Fig. 3), and (2) the differentially expressed genes plus their regulators and the selected endophenotypes (Supplementary Fig. 12).

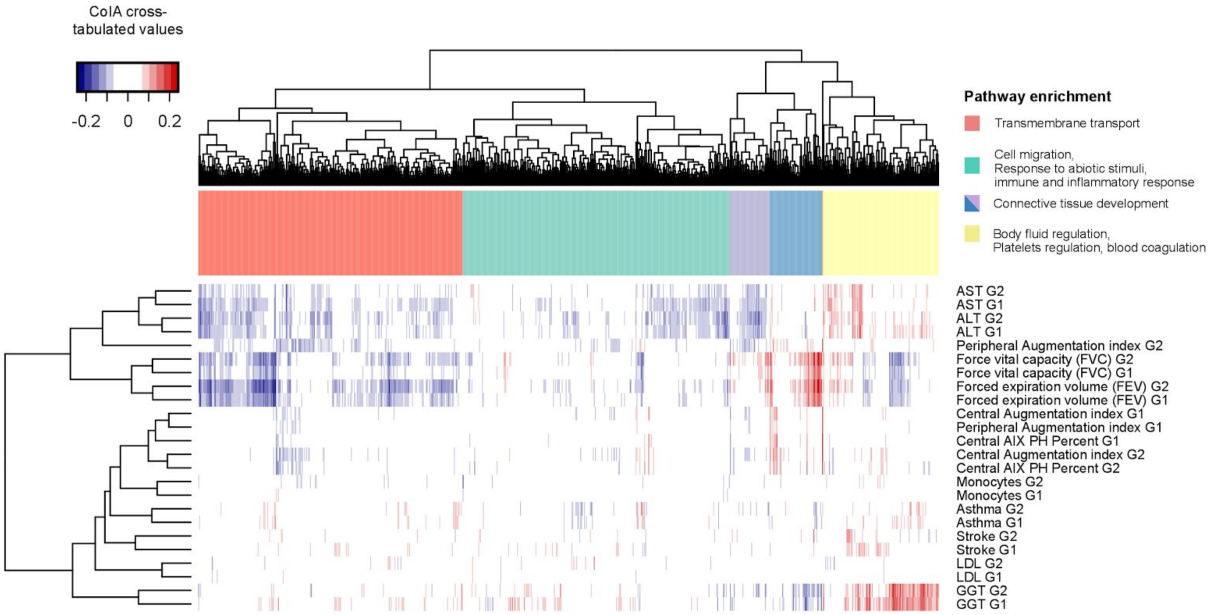

Supplementary Figure 12: Coinertia between the differentially expressed genes plus their regulators (RDEGs) and the selected endophenotypes.

Coinertia (CoIA) analysis between gene expression (columns) and their associated endophenotypes (rows). Genes were selected based on their differential expression among regions, or if they regulate the differentially expressed genes. The endophenotypes were the most associated with regional and fine scale environmental variables (Supplementary Fig. 8 and 9). Two sets of 500 of CoIAs were computed independently between gene expression and endophenotypes (Supplementary Fig. 11). The group 1 or 2 (G1 or G2) represents the freeze number 1 or 2, respectively. For each G1 or G2 a total of 1000 resampling of 493 individuals (without replacement) from the total population (n=997), were performed. The heatmap represents for each G1 or G2 the median of each endophenotype-gene association from the cross-tabulated values distribution. Phenotypes-expression associations from G1 and G2 largely cluster together, indicating a common signature of association. Detailed pathway enrichment results are given on Supplementary Fig. 13.

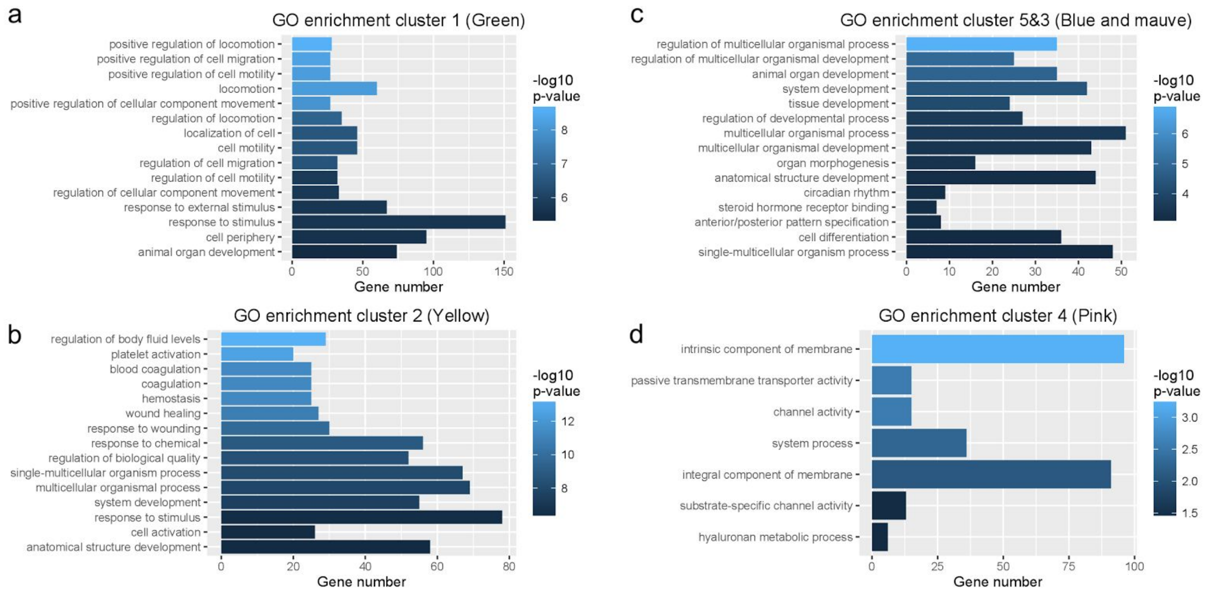

Supplementary Figure 13: Pathway enrichment from GO terms in gene clusters from CoIA between endophenotypes and differentially expressed genes (Fig. 3c).

Top 15 enriched terms are shown for each gene cluster identified by color annotation on columns of Supplementary Fig. 12. (a) Green cluster: enrichment for cell migration, immune and inflammatory response and response to abiotic stimuli. (b) Yellow cluster: enrichment in body fluid regulation, platelets regulation, and blood coagulation. (c) Blue and mauve cluster were analyzed together because of their close clustering. Enrichment in connective tissue development (d) Pink cluster: enrichment in transmembrane transport and channel activity.

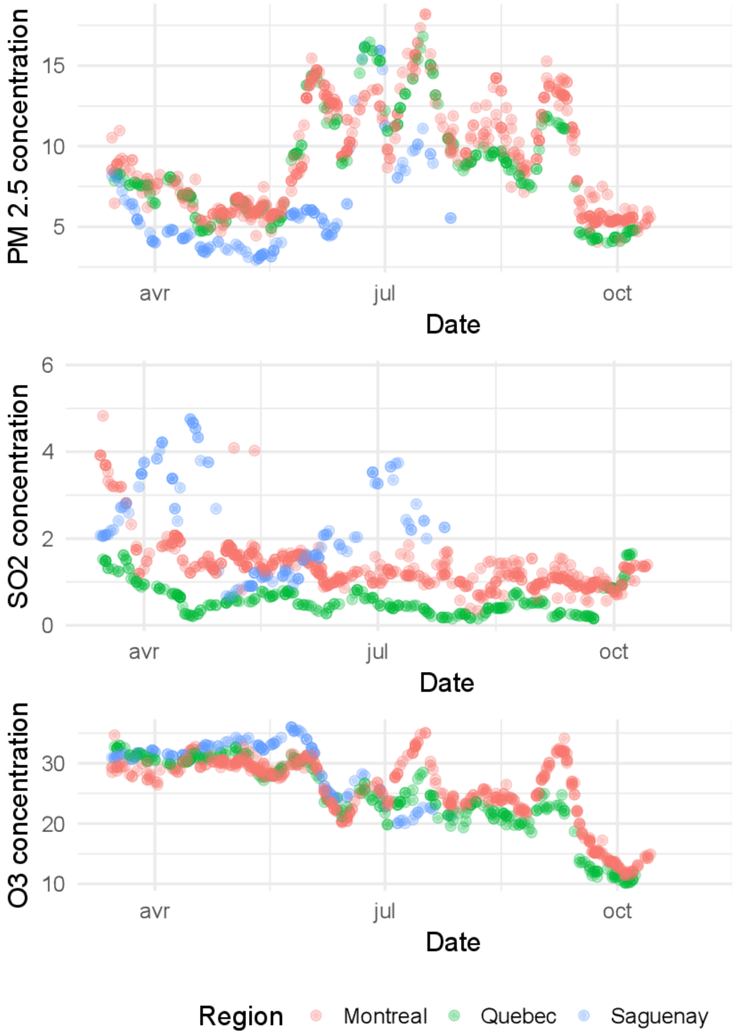

Supplementary Figure 14: Pollutant exposure for each CaG participant, 14-day average prior of day of blood sampling.

PM2.5, SO2 and O3 daily ambient levels (parts per billion) measured in each FSA were averaged for a 14-day period prior of blood sampling for each individual.

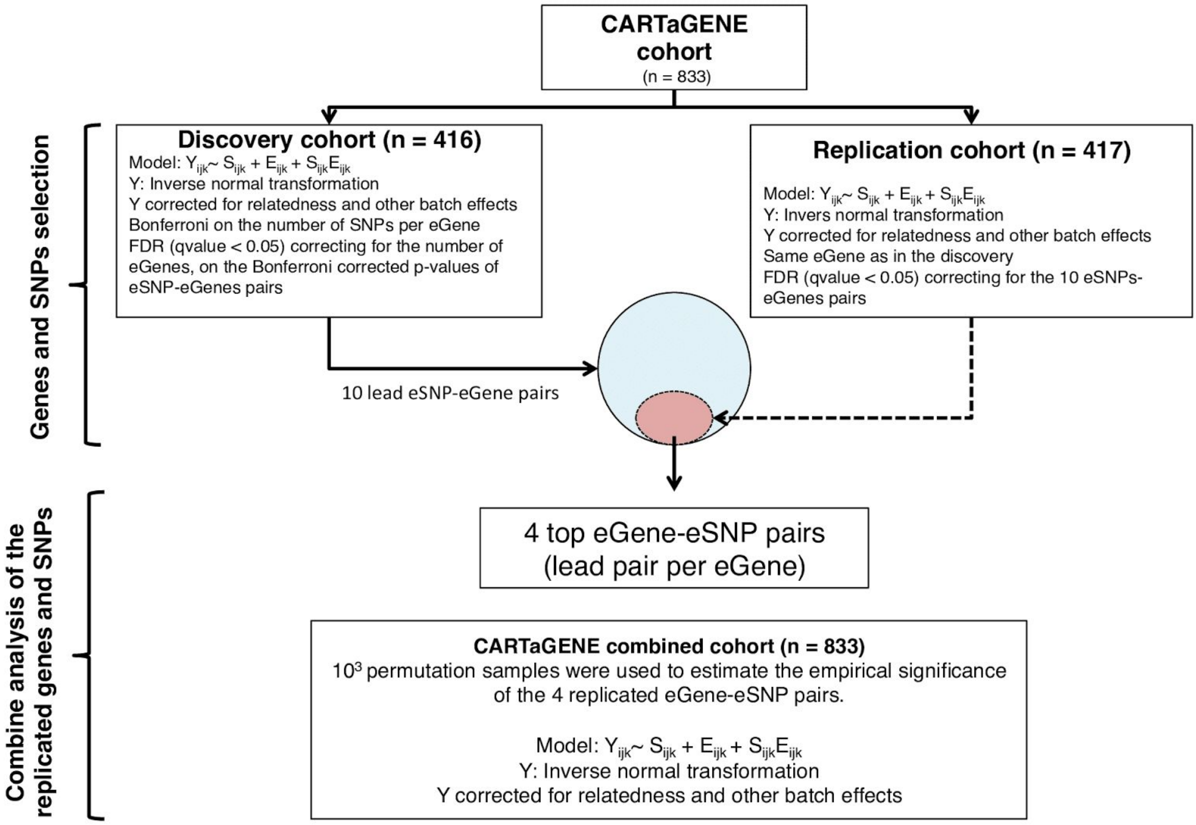

Supplementary Figure 15: Design and resampling scheme for env-eQTL analyses.

Pipeline to identify eQTLs for which the effect size is modulated by exposure with one of four ambient air pollutants (env-eQTLs): PM2.5, NO2, O3, and SO2. We retained the four eGene-eSNP pairs that replicated between the discovery and replication cohorts (with the same direction of effect), and assessed the empirical significance by generating 10^3 permutation samples from the combined cohort (details in the material and methods).

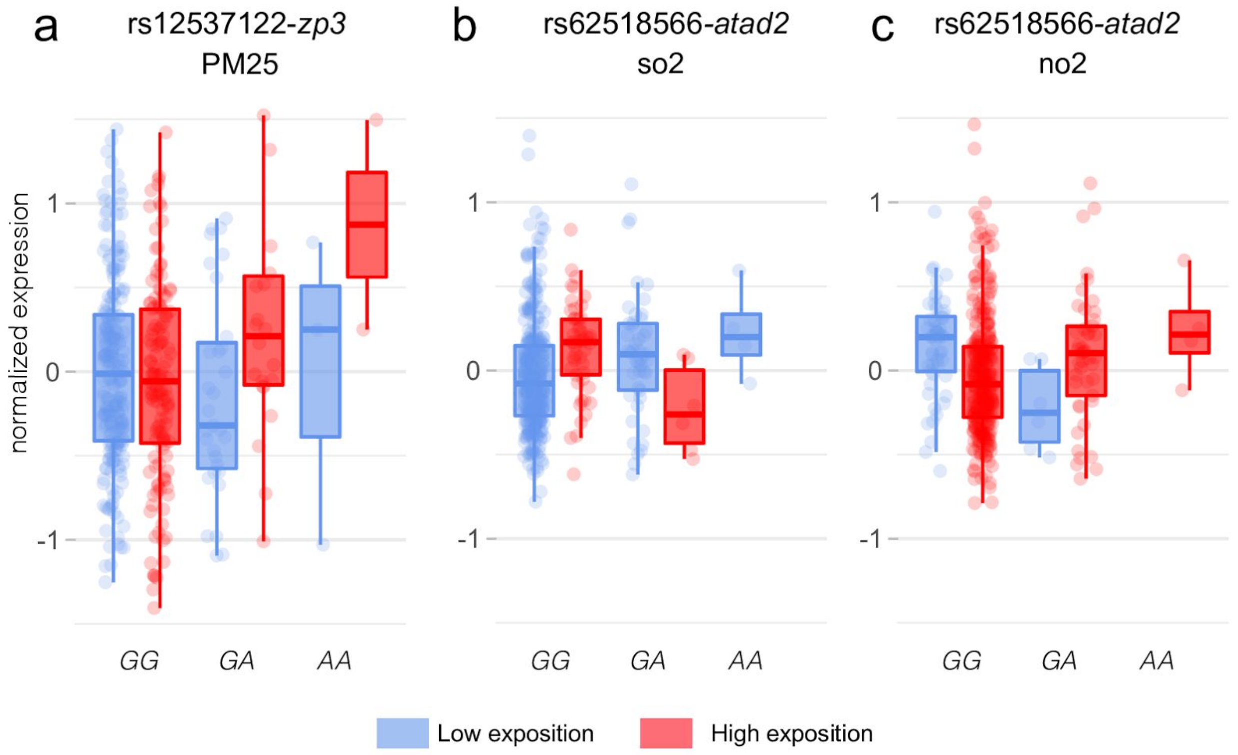

Supplementary Figure 16: Gene-environment interactions.

Top significant gene-environment interactions detected in CARTaGENE, represented as interaction graphs of env-eQTLs for the whole cohort. Red points (and boxplots) represent individuals exposed to a high level of the pollutant indicated in the title of each graph, and blue, represent low exposition to the pollutant. Genotype status of the eSNP is depicted on the x-axis and normalized expression of the eGene on the y-axis. (a) *zp3* expression is modulated by eSNP rs12537122 and PM2.5: ZP3 is a glycoprotein that has been linked to parkinson disease [3]. (b) *atad2* expression is modulated by eSNP rs62518566 and so2 and (c) no2. Interestingly, ATAD2 protein regulates chromatin dynamics and acts as a co-activator of estrogen and androgen receptors. It has been found that *atad2* is a marker of poor prognosis in a variety of different cancers [4,5]. The upper whiskers extend from the 3rd quartile to the largest value no further than 1.5 * inter-quartile range from the 3rd quartile. The lower whiskers extend from the first quartile to the smallest value at most 1.5 * inter-quartile range from the first quartile.

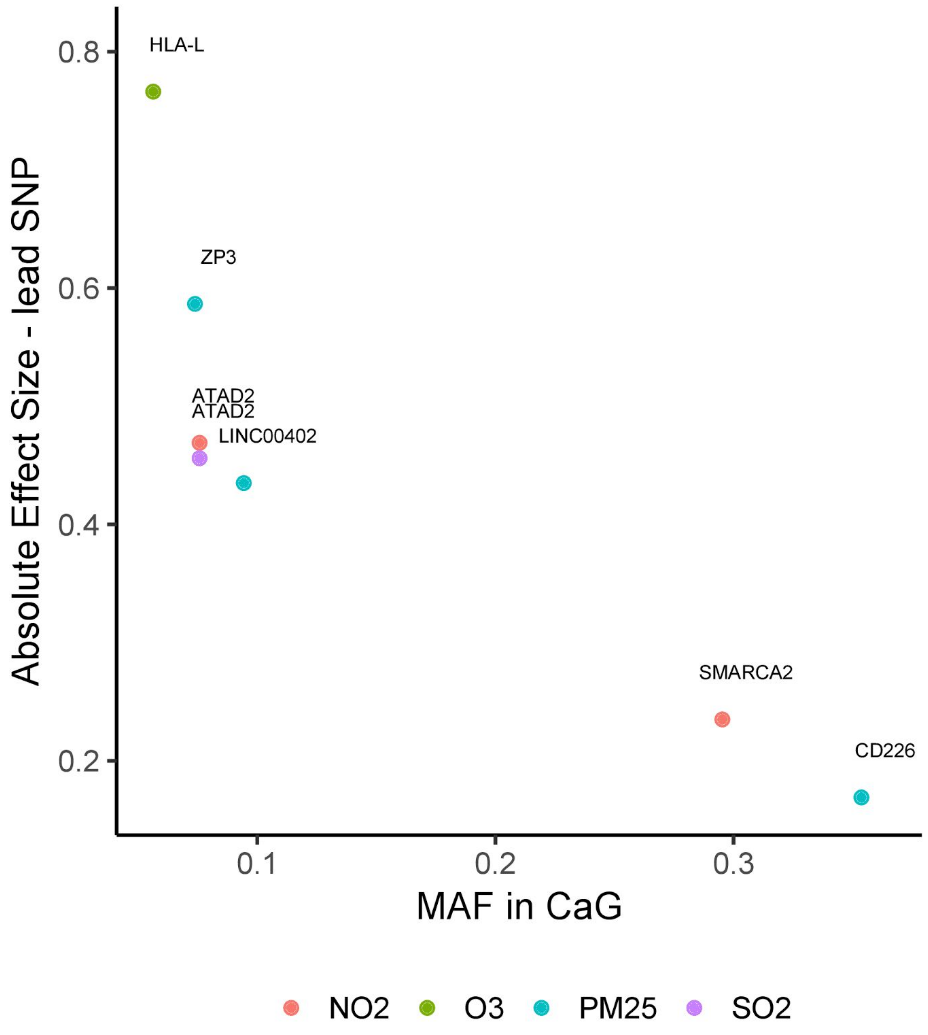

Figure S17: env-eQTL Effect sizes are larger for alleles with lower MAF
Relationship between MAF and effect sizes for env-eQTLs, for lead eSNPs. We observe an inverse relationship between absolute effect size and MAF. Extremes of gene expression in humans have been shown to associate with lower MAF [6] and burden of rare variants in cis-regulatory regions [7]. This is consistent with the hypothesis that transcript abundance is under stabilizing selection.

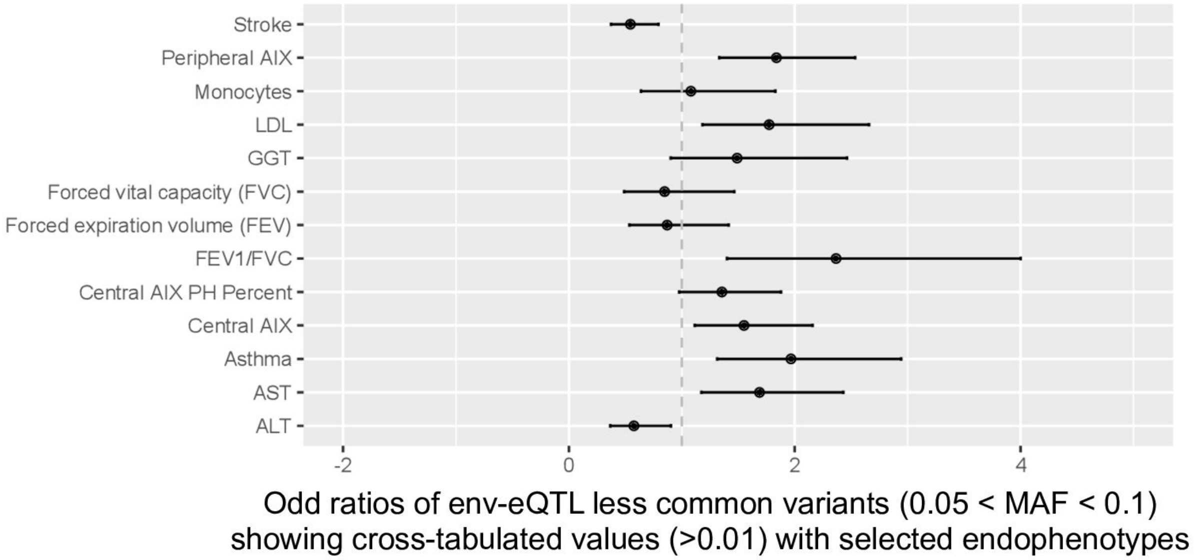

Supplementary Figure 18: Less common env-eQTL eSNPs are enriched for strong associations with endophenotypes

The odds ratio of observing less common variants (compared to common) for stronger endophenotypic associations is above 1 for FEV1/FVC ratio, central and peripheral AIX, Asthma, LDL and AST. We performed the CoIA analysis between all eSNPs (SNPs in cis of significant eGenes) and selected endophenotypic traits. The MAF was estimated from the data in the cohort, and we classified the SNPs as common (MAF > 0.1), and less common (MAF between 0.05 and 0.1). The 95% confidence intervals are depicted for each trait.

Supplementary tables

Supplementary Table 1: SVA correction removes most unwanted variation in gene expression profiles. Significant variables after a stepwise regression on expression profiles PC1 and PC2. Freeze 1 and Freeze 2 show a significant contribution of arterial stiffness, neutrophils counts, and Region to variation. Combining Freeze 1 and Freeze 2 together also show the same variables as significant, in addition to the Freeze. Once the total data has been corrected with SVA (n SVA=5) with specifically retaining the variation associated with region, the stepwise regression recovers region and Freeze as significant variables. Other biological variation that was significant before the SVA correction (Arterial Stiffness and neutrophils) has been efficiently removed. Freeze remains significant, and will be accounted for as a covariate in all subsequent analyses. The number of asterisks reflects the significance level of each variable in the stepwise regression. *=p<0.05,**=p<0.01,**=p<0.001,***=p<0.0001,****=p< 2.2 x 10^{-16}

	Freeze 1	Freeze 2	Freeze 1 and 2	SVA corrected - with variation around *Region* retained
PC1	Stiffness**<br>Neutrophil counts*	Region****	Freeze***<br>Region**<br>Neutrophil counts*<br>Stiffness*	Region***<br>Freeze**
PC2	Region***	Region***<br>Lymphocyte counts***<br>Neutrophil counts***	Region***<br>Freeze**	Region***<br>Freeze**

Supplementary Table 2: Distribution of locals and migrants across regions.

We identified the continental origin (French-Canadian or European) of each participant by combining the results of the principal component analysis on genotypes together with the self-reported origin of the grandparents. The regional ancestry of FC was determined based on the tree built from the chunck count matrix of chromopainter output.

**Origin**	**Residence**		
region or continent	Living in Mtl	Living in Que	Living in Sag
FC-Montreal	234	58	6
FC-Quebec	61	115	5
FC-Saguenay	34	41	115
European	108	16	12

Supplementary Table 3: Top differentially expressed genes between Mtl and Sag are enriched in oxygen, gas transport, Gpcr, and inflammatory response.

We performed a GO term enrichment on the top differentially expressed genes between Mtl and Sag (lfc > 0.65 and p-value < 0.05/15632, n=460).

GO ID	GO term	p-value
GO:0015671	oxygen transport	1.90E-07
GO:0015669	gas transport	3.40E-07
GO:0007267	cell-cell signaling	1.20E-06
GO:0007186	G-protein coupled receptor signaling pathway	1.30E-05
GO:0044057	regulation of system process	1.70E-05
GO:0006937	regulation of muscle contraction	3.20E-05
GO:0006954	inflammatory response	3.40E-05
GO:0006811	ion transport	0.00017
GO:0090257	regulation of muscle system process	0.00017
GO:0070588	calcium ion transmembrane transport	0.00018
GO:0048673	collateral sprouting of intact axon in response to injury	0.00023
GO:0048683	regulation of collateral sprouting of intact axon in response to injury	0.00023
GO:0051480	cytosolic calcium ion homeostasis	0.00028
GO:0098655	cation transmembrane transport	0.00029
GO:0034220	ion transmembrane transport	0.00036
GO:0055085	transmembrane transport	0.00039
GO:0098660	inorganic ion transmembrane transport	0.0005
GO:0044707	single-multicellular organism process	0.00054
GO:0006874	cellular calcium ion homeostasis	0.0006
GO:0006942	regulation of striated muscle contraction	0.00066
GO:0055074	calcium ion homeostasis	0.00077

GO:0042044	fluid transport	0.00082
GO:0032501	multicellular organismal process	0.00084
GO:0006935	chemotaxis	0.00095
GO:0042330	taxis	0.00095
GO:0072503	cellular divalent inorganic cation homeostasis	0.00098
GO:0007268	synaptic transmission	0.00102
GO:0034765	regulation of ion transmembrane transport	0.00112
GO:0006941	striated muscle contraction	0.00123
GO:0006936	muscle contraction	0.00126
GO:0060326	cell chemotaxis	0.00127
GO:0033280	response to vitamin D	0.00141
GO:0072507	divalent inorganic cation homeostasis	0.00141
GO:0034762	regulation of transmembrane transport	0.00161
GO:0098662	inorganic cation transmembrane transport	0.00174
GO:0040011	locomotion	0.00202
GO:0031102	neuron projection regeneration	0.00206
GO:0003008	system process	0.00206
GO:0042476	odontogenesis	0.00221
GO:0031282	regulation of guanylate cyclase activity	0.00227
GO:0045933	positive regulation of muscle contraction	0.00244
GO:0015701	bicarbonate transport	0.00286
GO:0052652	cyclic purine nucleotide metabolic process	0.00314
GO:0009190	cyclic nucleotide biosynthetic process	0.00336
GO:0007168	receptor guanylyl cyclase signaling pathway	0.00337
GO:0014819	regulation of skeletal muscle contraction	0.00337
GO:0030826	regulation of cGMP biosynthetic process	0.00337
GO:0007204	positive regulation of cytosolic calcium ion concentration	0.00384
GO:0030049	muscle filament sliding	0.00384
GO:0033275	actin-myosin filament sliding	0.00384

Supplementary Table 4

Variable	Data source
**Air pollution**	
14 day SO2 community levels (ppb): average concentration measured at NAPS stations ≤25 km from FSA centroid	Canadian National Air Pollutant Surveillance Network (NAPS) : http://www.ec.gc.ca/rnspa-naps/default.asp?lang=En&n=5C0D33CF
14 day O3 community levels (ppb): average concentration measured at NAPS stations ≤25 km from FSA centroid	Canadian National Air Pollutant Surveillance Network (NAPS) : http://www.ec.gc.ca/rnspa-naps/default.asp?lang=En&n=5C0D33CF
14 day PM2.5 community levels (µg/m3): average concentration measured at NAPS stations ≤25 km from FSA centroid	Canadian National Air Pollutant Surveillance Network (NAPS) : http://www.ec.gc.ca/rnspa-naps/default.asp?lang=En&n=5C0D33CF
Annual fine scale NO2 levels (ppb): model resolution ≤1 km	Satellite-LUR with empirical adjustment: Hystad et al. 2011 (https://ehp.niehs.nih.gov/1002976).
Annual fine scale PM2.5 levels (µg/m3): model resolution ≤1 km	Geographically weighted satellite-LUR: Van Donkelaar et al. 2015 (http://pubs.acs.org/doi/abs/10.1021/acs.est.5b02076)<br>Van Donkelaar e al. 2010 (https://ehp.niehs.nih.gov/0901623/)<br><br>data available at :<br>http://fizz.phys.dal.ca/~atmos/martin/?page_id=140
**Built environment**	
Street network	https://www.inspq.qc.ca/expertises/habitudes-de-vie-et-prevention-des-maladies-chroniques/nutrition-activite-physique-et-problemes-lies-au-poids/portrait-de-l-environnement-bati-et-de-l-environnement-des-services/indicateurs-de-l-environnement-bati/connexite-du-reseau-routier
Population density	https://www.inspq.qc.ca/expertises/habitudes-de-vie-et-prevention-des-maladies-chroniques/nutrition-activite-physique-et-problemes-lies-au-poids/portrait-de-l-environnement-bati-et-de-l-environnement-des-services/indicateurs-de-l-environnement-bati/densite-residentielle

Food desert	https://www.inspq.qc.ca/expertises/habitudes-de-vie-et-prevention-des-maladies-chroniques/nutrition-activite-physique-et-problemes-lies-au-poids/portrait-de-l-environnement-bati-et-de-l-environnement-des-services/environnement-des-services/indice-de-l-environnement-alimentaire
Park density (number of parks by area)	https://www.inspq.qc.ca/expertises/habitudes-de-vie-et-prevention-des-maladies-chroniques/nutrition-activite-physique-et-problemes-lies-au-poids/portrait-de-l-environnement-bati-et-de-l-environnement-des-services/environnement-des-services/mesure-de-distance-parcs-et-espaces
Greenness	https://www.inspq.qc.ca/expertises/habitudes-de-vie-et-prevention-des-maladies-chroniques/nutrition-activite-physique-et-problemes-lies-au-poids/portrait-de-l-environnement-bati-et-de-l-environnement-des-services/indicateurs-de-l-environnement-bati/design-de-l-environnement
Walkability	https://www.inspq.qc.ca/expertises/habitudes-de-vie-et-prevention-des-maladies-chroniques/nutrition-activite-physique-et-problemes-lies-au-poids/portrait-de-l-environnement-bati-et-de-l-environnement-des-services/indicateurs-de-l-environnement-bati/indice-de-potentiel-pietonnier
**Socioeconomic status (SES)**	
Social and material deprivation indexes	https://www.inspq.qc.ca/expertises/habitudes-de-vie-et-prevention-des-maladies-chroniques/nutrition-activite-physique-et-problemes-lies-au-poids/portrait-de-l-environnement-bati-et-de-l-environnement-des-services/indice-de-defavorisation

Supplementary Table 5: Description of the 57 clinically relevant endophenotypes.
These endophenotypes were selected to encompass most body systems that characterize health in adult humans. Only endophenotypes with sufficient individuals reporting the condition were included. Some lifestyle measures (alcohol consumption, smoking habits, daily vegetable intake) were also included to verify their possible correlation with region. None of those lifestyle habits are different across regions or environments.

Endophenotype	Description
Age	Age
Alcohol consumption	Healthy / Heavy
Allergy	Yes / No
ALT (liver function)	Alanine aminotransferase level
AST (liver function)	Aspartate aminotransferase level
Asthma	Yes / No
Augmentation index PH percent	Measure of wave reflection and arterial stiffness, measured with SphygmoCor
BMI	Body mass index
Bone density	T-score bone density
Bronchitis	Yes / No
Buckberg ratio	Subendocardial viability ratio (index of myocardial oxygen supply and demand)
Cancer	Yes / No
Central Augmentation Index	Measure of wave reflection and arterial stiffness, measured with SphygmoCor
Chronic Renal problem	Yes / No
Daily vegetables	Good / Poor
Diastolic BP	Diastolyic blood pressure
Ejection duration	Duration of heart ventricular ejection

Emphysema	Emphysema diagnostic - Yes or No
Eosinophils	Eosinophils counts
Erythrocytes	Erythrocytes counts
Fat percent	Fat percent from body impedence
FEV1 to FVC ratio	Spirometry
Forced expiratory volume (FEV1)	Spirometry
Forced vital capacity (FVC)	Spirometry
Gender	Male / Female
GGT (liver function)	Gamma-glutamyl transferase levels
HBA1C	Glycated heamoglobin levels
HDL	High density lipoprotein level
Heart	Heart problem diagnosed - Yes / No
Height	Height (cm)
Hematocrit	Hematocrit blood levels
Hemoglobin	Hemoglobin blood levels
LDL	Low density lipoprotein
Leukocytes	Leukocytes counts
Lung	Lung problem diagnosed - Yes / No
Lymphocytes	Lymphocytes counts
Macular degeneration	Yes / No
Mean corpuscular volume	Mean corpuscular volume
Mean CV conc	Mean corpuscular volume concentration
Mean platelet volume	Mean platelet volume
Monocytes	Monocytes counts
# Chronic Diseases	1 to 5
Neutrophils	Neutrophils counts
Peripheral augmentation index	Measure of wave reflection and arterial stiffness,

	measured with SphygmoCor
Physical activity	IPAQ score category
Platelets	Platelets counts
Red blood cell distribution	Red blood cell distribution width
Resting heat rate	Heart rate at rest
Skin	Skin problem diagnosed - Yes / No
Smoking status	Yes, Past, Occasional, Never
Stiffness score	Derived from pulse wave analysis (Low, medium, high)
Stroke	Yes / No
Systolic BP	Systolic Blood pressure
T2 Diabetes	Yes / No
T4 (Thyroid)	T4 levels
Triglycerides	Triglycerides levels
TSH (Thyroid)	Thyroid stimulationg hormone levels

Supplementary Table 6: Differentially expressed genes between high and low SO2 exposure are enriched in oxygen transport, chemokine activity, Gpcr, and leukocyte migration pathways.

We performed a GO term enrichment on the 170 differentially expressed genes between high and low SO2 exposure.

GO.ID	Term	pValue
GO:0019825	oxygen binding	1.09E-08
GO:0005833	hemoglobin complex	1.65E-08
GO:0070098	chemokine-mediated signaling pathway	2.32E-07
GO:0005344	oxygen transporter activity	8.95E-07
GO:0015671	oxygen transport	2.21E-06
GO:0045236	CXCR chemokine receptor binding	4.81E-06
GO:0007186	G-protein coupled receptor signaling pathway	7.14E-06
GO:0015669	gas transport	9.52E-06
GO:0005125	cytokine activity	2.99E-05
GO:0008009	chemokine activity	4.99E-05
GO:0030595	leukocyte chemotaxis	0.000107
GO:0005615	extracellular space	0.000146
GO:0060326	cell chemotaxis	0.000219
GO:0009617	response to bacterium	0.000358
GO:0042379	chemokine receptor binding	0.00092
GO:0002687	positive regulation of leukocyte migration	0.00122
GO:0009605	response to external stimulus	0.00213
GO:0002685	regulation of leukocyte migration	0.00313
GO:0006954	inflammatory response	0.00379
GO:0002688	regulation of leukocyte chemotaxis	0.00428
GO:0050900	leukocyte migration	0.00445

GO:0031091	platelet alpha granule	0.0057
GO:0006935	chemotaxis	0.00655
GO:0042330	taxis	0.00674
GO:0032496	response to lipopolysaccharide	0.01
GO:0060205	cytoplasmic membrane-bounded vesicle lumen	0.01
GO:0031983	vesicle lumen	0.0115
GO:0002690	positive regulation of leukocyte chemotaxis	0.0131
GO:0005576	extracellular region	0.0153
GO:0031224	intrinsic component of membrane	0.0154
GO:0004930	G-protein coupled receptor activity	0.0156
GO:0002237	response to molecule of bacterial origin	0.0159
GO:0016021	integral component of membrane	0.0182
GO:0043207	response to external biotic stimulus	0.0253
GO:0051707	response to other organism	0.0253
GO:0045765	regulation of angiogenesis	0.0295
GO:0020037	heme binding	0.0302
GO:0009607	response to biotic stimulus	0.0493

Supplementary Table 7: Multivariate models show that 14-day SO2 air pollution contributes to modulating gene expression in 170 gene candidates, and not most covariates or underlying diseases.

We tested for the contribution of several environmental variables (models 1,2 and 5,6) to variation in gene expression in the 170 candidates identified through differential gene expression analyses. We performed sensitivity analyses by restricting our samples to MTL-only, thereby reducing the possible effect of other variables associated with geography and regional ancestry. We also tested for the contribution of associated underlying diseases/phenotypic states (models 3 and 4) on gene expression variation. Models 5 and 6 (*diseases removed*) are performed on gene expression data form which the effects of FEV1, liver enzymes, lung diseases and arterial stiffness have been removed (significant phenotypes in Model 3). Details on phenotypes are given in Supplementary Table 5.

Significance levels: *=$p<0.05$,**=$p<0.01$,**=$p<0.001$,***=$p<0.0001$,****=$p<2.2x10^{-16}$

Model 1	gene expression ALL SO2** + O3** + PM2.5 + smoking + Population density* + greenness** + social deprivation + long term NO2 + food desert + park density + street network + material deprivation* + walkability + long term PM2.5
Model 2	gene expression MTL-only SO2** + O3* + PM2.5 + smoking + Population density + greenness* + social deprivation + long term NO2 + food desert + park density + street network* + material deprivation + walkability + long term PM2.5
Model 1 and 2 replication	SO2 + O3 + greenness
Model 3	gene expression ALL Lung** + Asthma + AST*** + ALT*** + Arterial stiffness* FEV1*** + lymphocyte counts + monocyte counts + GGT*
Model 4	gene expression MTL-only Lung + Asthma + AST + ALT + Arterial stiffness FEV1*** + lymphocyte counts + monocyte counts + GGT

Model 3 and 4 replication	FEV1
Model 5<br><br><br><br><br><br>Model 6	gene expression *diseases removed* ALL SO2** + O3 + PM2.5 + smoking + Population density* + greenness*** + social deprivation + long term NO2 + food desert + park density + street network + material deprivation* + walkability + long term PM2.5<br><br>gene expression *diseases removed* MTL-only SO2** + O3 + PM2.5 + smoking + Population density + greenness + social deprivation + long term NO2 + food desert + park density + street network + material deprivation + walkability + long term PM2.5
Model 5 and 6 replication	SO2

Supplementary Table 8: Canonical eQTLs in large scale studies replicate in CARTaGENE.

Features identified in canonical eQTLs (eSNPs, SNP-gene pairs, eGenes) in other large scale studies (Geuvadis, Hap Map, and GTEx (blood)) replicate with high proportion in CARTaGENE French-Canadians. The highest replication rate is acheived with whole blood samples from GTEx. The other two studies were based on cell lines. Numbers are percents of replication.

Feature	Geuvadis	Hap Map	GTEx-blood
eSNPs	86.9	77.6	96.3
SNP-gene pairs	78.6	69.7	93.6
eGenes	78	64.7	93.8

Supplementary Table 9: Interaction env-eQTLs

Replicated eSNP-eGene pairs of env-eQTLs in discovery and replication cohorts, and in the combined dataset. All entries are significant (q-value<0.05; see Material and Methods) with the same direction of effect in both the discovery and replication cohorts, and remain significant through empirical estimation of p-values using permutation in the combined cohort.

					Discovery (n=416)			Replication (n=417)			Combined (n=833)			
eSNP	Effect Allele	MAF	eGene	Pollutant	Effect Size	SE	p-value	Effect Size	SE	p-value	Effect Size	SE	p-value	Empirical p-value (permutations)
**rs10156534**	**G**	**0.29530**	**SMARCA2**	**NO2**	**-1.770**	**0.276**	**3.42e-10**	**-0.235**	**0.081**	**0.0035**	**-0.890**	**0.425**	**0.0364**	**0.002**
**rs62518566**	**G**	**0.07576**	**ATAD2**	**NO2**	**1.021**	**0.153**	**7.98e-11**	**0.469**	**0.163**	**0.0035**	**0.519**	**0.248**	**0.0369**	**0.005**
**rs12537122**	**A**	**0.07384**	**ZP3**	**PM25**	**0.566**	**0.109**	**3.13e-7**	**0.587**	**0.169**	**0.0006**	**0.293**	**0.097**	**0.0013**	**0.035**
**rs62518566**	**A**	**0.07576**	**ATAD2**	**SO2**	**-1.804**	**0.278**	**3.94e-10**	**-0.455**	**0.155**	**0.0035**	**-0.914**	**0.425**	**0.0316**	**0.001**

Supplementary references

1. *10 years of data from the national air pollution surveillance (NAPS) network*. (Goverment of Canada, 2013).
2. Wood, J. *Canadian Environmental Indicators - Air Quality*. (Fraser Institute, 2012).
3. Beecham, G. W. *et al.* PARK10 is a major locus for sporadic neuropathologically confirmed Parkinson disease. *Neurology* **84,** 972–980 (2015).
4. Ciró, M. *et al.* ATAD2 is a novel cofactor for MYC, overexpressed and amplified in aggressive tumors. *Cancer Res.* **69,** 8491–8498 (2009).
5. Wu, G. *et al.* Epigenetic high regulation of ATAD2 regulates the Hh pathway in human hepatocellular carcinoma. *Int. J. Oncol.* **45,** 351–361 (2014).
6. Battle, A. *et al.* Characterizing the genetic basis of transcriptome diversity through RNA-sequencing of 922 individuals. *Genome Res.* **24,** 14–24 (2014).
7. Zhao, J. *et al.* A Burden of Rare Variants Associated with Extremes of Gene Expression in Human Peripheral Blood. *Am. J. Hum. Genet.* **98,** 299–309 (2016).
